# The Cyclin-Dependent Kinase Ortholog pUL97 of Human Cytomegalovirus Interacts with Cyclins

**DOI:** 10.3390/v5123213

**Published:** 2013-12-18

**Authors:** Laura Graf, Rike Webel, Sabrina Wagner, Stuart T. Hamilton, William D. Rawlinson, Heinrich Sticht, Manfred Marschall

**Affiliations:** 1 Institute for Clinical and Molecular Virology, University of Erlangen-Nuremberg, 91054 Erlangen, Germany; E-Mails: graf.laura@gmx.de (L.G.); rike.webel@viro.med.uni-erlangen.de (R.W.); sabrina.wagner@viro.med.uni-erlangen.de (S.W.); 2 Virology Division, SEALS, Department of Microbiology, Prince of Wales Hospital, Randwick, NSW 2031, Sydney, Australia; E-Mails: z3058477@zmail.unsw.edu.au (S.T.H.); w.rawlinson@unsw.edu.au (W.D.R.); 3 Division of Bioinformatics, Institute of Biochemistry, University of Erlangen-Nuremberg, 91054 Erlangen, Germany; E-Mail: Heinrich.Sticht@med.uni-erlangen.de

**Keywords:** human cytomegalovirus, protein kinase pUL97, cyclins T1, B1 and A, protein-protein interaction, substrate phosphorylation, interaction-mediated regulation

## Abstract

The human cytomegalovirus (HCMV)-encoded protein kinase, pUL97, is considered a cyclin-dependent kinase (CDK) ortholog, due to shared structural and functional characteristics. The primary mechanism of CDK activation is binding to corresponding cyclins, including cyclin T1, which is the usual regulatory cofactor of CDK9. This study provides evidence of direct interaction between pUL97 and cyclin T1 using yeast two-hybrid and co-immunoprecipitation analyses. Confocal immunofluorescence revealed partial colocalization of pUL97 with cyclin T1 in subnuclear compartments, most pronounced in viral replication centres. The distribution patterns of pUL97 and cyclin T1 were independent of HCMV strain and host cell type. The sequence domain of pUL97 responsible for the interaction with cyclin T1 was between amino acids 231–280. Additional co-immunoprecipitation analyses showed cyclin B1 and cyclin A as further pUL97 interaction partners. Investigation of the pUL97-cyclin T1 interaction in an ATP consumption assay strongly suggested phosphorylation of pUL97 by the CDK9/cyclin T1 complex in a substrate concentration-dependent manner. This is the first demonstration of interaction between a herpesviral CDK ortholog and cellular cyclins.

## 1. Introduction

Human cytomegalovirus (HCMV), also known as human herpesvirus 5 (HHV-5), is a member of the β-*Herpesvirinae* subfamily. It is a ubiquitous human pathogen of increasing seroprevalence in different populations (60%–90%) that causes severe systemic diseases in immunosuppressed patients and is the leading infectious cause of birth defects in developed countries [1]. Currently approved antiviral agents for systemic treatment (cidofovir, foscarnet, ganciclovir and valganciclovir) inhibit viral DNA synthesis by targeting the HCMV DNA polymerase, pUL54 [2]. However, drug-resistant virus variants emerge after prolonged therapy, and current antivirals cause frequent adverse side effects. Protein kinases are putative targets of novel antiviral drugs, given their important role in the regulation of HCMV replication [3,4,5,6,7,8]. Pharmacological cyclin-dependent kinase (CDK) inhibitors interfere with the replication of HCMV and other viruses and are currently being investigated in a number of clinical trials. Roscovitine, a purine analogue that preferentially inhibits CDK1, 2, 5, 7 and 9, has been shown to decrease viral DNA synthesis and production of late viral proteins and infectious virus [4]. Recently, we reported that a novel selective CDK9 inhibitor, R22, exerts anti‑cytomegaloviral activity in cell culture models [9]. CDKs are heterodimeric serine/threonine kinases phosphorylating a number of substrate proteins. Upon activation through binding to their regulatory cyclin subunits, CDKs regulate cell cycle progression, transcription, neuronal cytoskeleton organization, apoptosis and other cellular functions. These kinases are promising targets for anti-cytomegaloviral therapy, since the efficiency of HCMV replication is closely connected to CDK activity [4,8,9,10,11]. Moreover, HCMV is able to stimulate or suppress CDK activity in order to create an environment favourable for efficient viral transcription, genome replication and assembly of viral particles. At least four CDKs (CDK1, 2, 7 and 9) and their corresponding cyclins are required for efficient HCMV replication and are upregulated in HCMV-infected cells [3,12,13,14,15].

**Figure 1:**
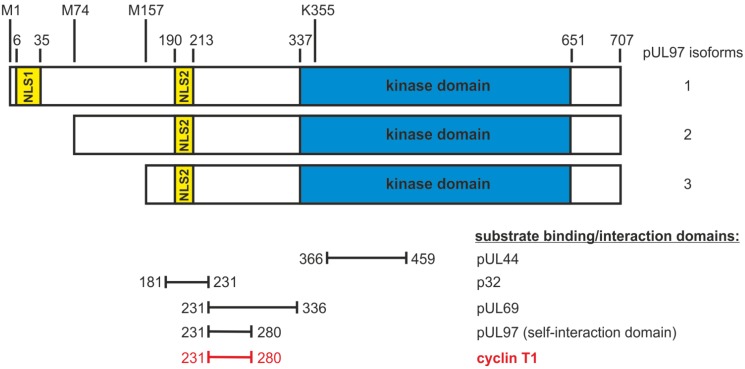
Schematic illustration of the structure of pUL97. Kinase domain (amino acids 337–651): subdivided into 11 subdomains (SD) that are conserved within herpesviral protein kinases and are similarly found in cellular protein kinases; mutation of the invariant lysine residue of SD II (amino acid 355) results in a complete loss of kinase activity [18,23,24]. Expression of three pUL97 isoforms: formation by the alternative initiation of translation with start codons at amino acids 1, 74 and 157 [25,26,27]. Nuclear localization signals (NLS1 amino acids 6–35 and NLS2 amino acids 190–213): mediation of the nuclear import of pUL97 isoforms by importin-α binding [26,28]. Self‑interaction domain of pUL97 (amino acids 231–280): formation of dimers and oligomers [29]. Regulation of various processes during human cytomegalovirus (HCMV) replication by pUL97 via phosphorylation of viral and cellular substrates: protein synthesis (RNA polymerase II [30], EF-1δ [18,31], pUL69 [32]), viral DNA replication (pUL44 [33]), nuclear capsid egress (lamins A and C, p32, a multi-ligand binding protein, also referred to as gC1qR and HABP1 [34,35], morphogenesis (pp65 [36]), cell cycle modulation (Rb [20]) and antiviral therapy (ganciclovir [6]).

HCMV not only modulates CDK regulation of the host cell, but also mimics CDK activity through expression of the serine/threonine protein kinase, pUL97. HCMV pUL97 is considered a CDK ortholog, due to structural and functional similarities. Although pUL97 does not appear to be absolutely required for viral replication, deletion of the ORF UL97 from the viral genome or pharmacological inhibition of pUL97 significantly reduces virus replication, showing the importance of pUL97 activity for efficient virus replication [16,17]. pUL97 regulates HCMV at various stages of replication by phosphorylating viral and cellular proteins (Figure 1). Sequence analyses and a three‑dimensional pUL97 model suggested conservation of functionally important residues in ATP binding sites and the catalytic centre between pUL97 and CDKs [18,19]. Recently, it has been reported that pUL97 phosphorylates cellular retinoblastoma protein (Rb) at the same residues as CDKs, a protein which controls progression through the G1 phase of the cell cycle [20,21]. There are further shared substrates of CDKs and pUL97, including nuclear lamins A and C, RNA polymerase II and pUL69 (Figure 1). In addition, inhibition of CDKs potentiates the effect of the pUL97 inhibitor, maribavir, indicating that the functions of CDKs and pUL97 overlap to some extent [22]. Moreover, a yeast complementation assay demonstrated a pUL97-mediated rescue of the proliferation of a *Saccharomyces cerevisiae* mutant lacking CDK activity [20]. It has been suggested that pUL97 is regulated in a cyclin-independent manner, a conclusion based on the finding that cyclins did not copurify with pUL97 during tandem affinity purification, so that binding of pUL97 to cyclins was considered unlikely [20]. However, in the present study, we provide the first evidence for the interaction of pUL97 with cyclins, emphasizing the functional relation between the viral CDK ortholog pUL97 and cellular CDKs. The meaning of these interactions for phosphorylation-dependent regulatory processes during HCMV replication and possible functional consequences are discussed. 

## 2. Results

### 2.1. Interaction of the HCMV Protein Kinase pUL97 with Cyclin T1

**Figure 2:**
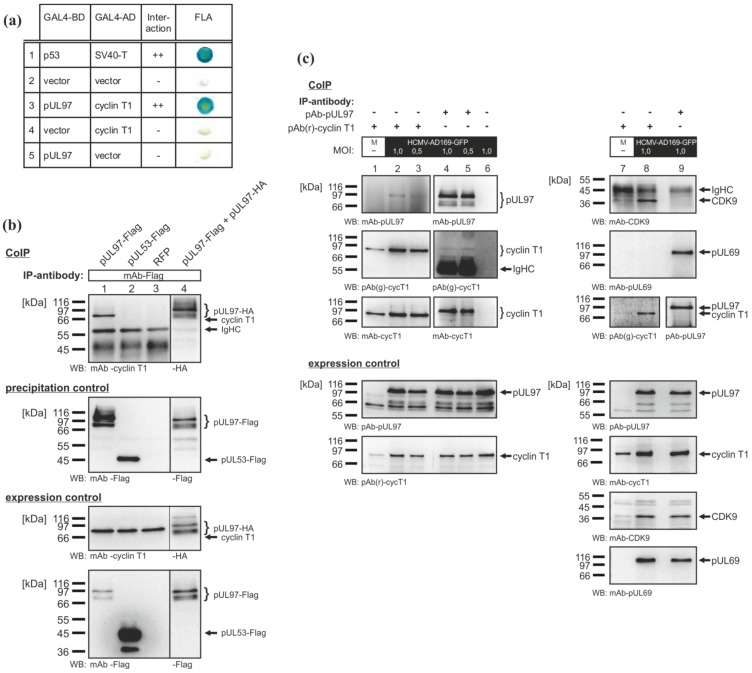
Direct interaction between pUL97 and cyclin T1. (**a**) Yeast two-hybrid (Y2H) analysis: cyclin T1 fused to the GAL4 activation domain (AD) and pUL97 fused to the GAL4 binding domain (BD) were coexpressed in yeast cells, and colonies were stained in filter lift assays (FLA). (**b**) co-immunoprecipitation (CoIP) analyses using protein lysates of transfected 293T cells: the self-interaction of pUL97 served as a positive control (lane 4) and pUL53-Flag as a negative control (lane 2). (**c**) CoIP analyses showing the interaction between pUL97 and cyclin T1 in HCMV-infected cells: human foreskin fibroblasts (HFFs) were infected with HCMV AD169-GFP at a multiplicity of infection (MOI) of 0.5 or 1.0. At six days post-infection, infected and mock-infected (M) cells were lysed and used for CoIP analyses. IP, immunoprecipitation; WB, Western blot analysis.

Yeast two-hybrid (Y2H) experiments revealed the interaction between pUL97 and cyclin T1 (Figure 2a, row 3). The signal obtained in the filter lift assay was similar to the positive control (interaction between p53 and SV40 T-antigen, Figure 2a, row 1). This was considered specific, since the intrinsic autoactivation effects of the individual fusion proteins were excluded by vector controls (Figure 2a, rows 4 and 5). The interaction between pUL97 and cyclin T1 was confirmed using co-immunoprecipitation (CoIP) analyses with protein lysates from transiently transfected 293T cells (Figure 2b). Endogenous cyclin T1 was specifically co-immunoprecipitated together with Flag-tagged pUL97 (Figure 2b, lane 1). Positive controls (pUL97 self-interaction, Figure 2b, lane 4) and negative controls (expression of RFP and pUL53, respectively, Figure 2b, lane 2, 3) confirmed the reliability of signals. Further CoIP analyses also demonstrated the pUL97-cyclin T1 interaction in HCMV-infected human foreskin fibroblast (HFF) cells (Figure 2c). pUL97 was co-immunoprecipitated with cyclin T1 (Figure 2c, lanes 2 and 3) and *vice versa* (Figure 2c, lanes 4 and 5). The intensity of the detected signals was independent of multiplicity of infection, when tested at multiplicity of infection (MOI) 0.5 and 1.0. The known interactions between cyclin T1 and CDK9 [37], as well as between pUL97 and the viral mRNA export factor, pUL69 [32], served as positive controls (Figure 2c, lanes 7–9). The weak signal of co-immunoprecipitated CDK9 in uninfected cells was a result of low expression and subsequent limited immunoprecipitation of cyclin T1. Interestingly, CDK9 did not co‑immunoprecipitate with pUL97 (Figure 2c, lane 9), whereas both proteins were detected in the precipitate of cyclin T1. These findings provide the first evidence of the interaction of a herpesviral kinase with a cellular cyclin.

### 2.2. Partial Colocalization of pUL97 with Cyclin T1 in Subnuclear Compartments of HCMV-Infected Cells

**Figure 3:**
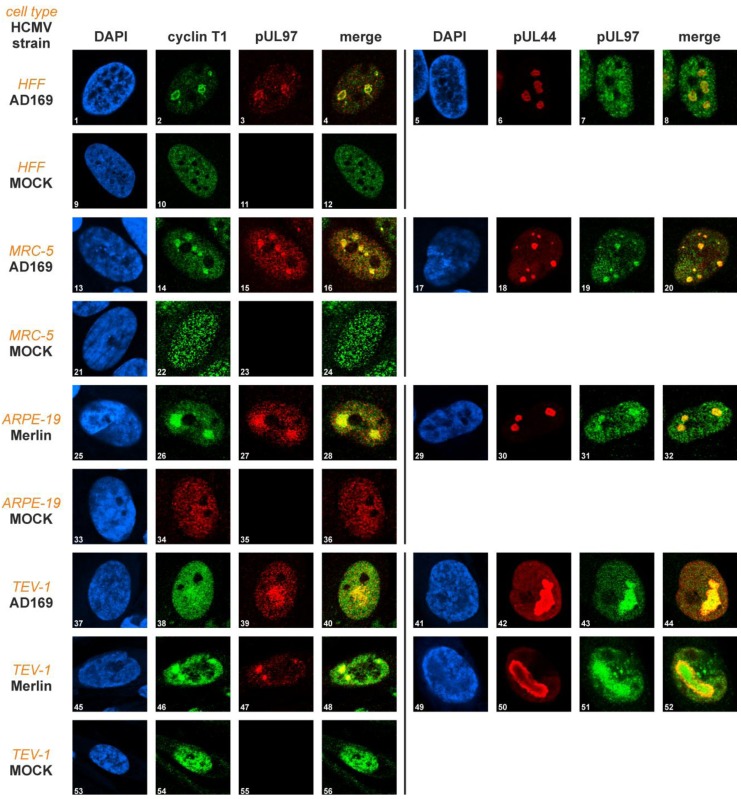
Comparison of the pUL97-cyclin T1 intracellular localization in four human cell types infected with two different HCMV strains using immunofluorescence analyses. HFF, MRC-5, ARPE-19 and TEV-1 cells were infected with HCMV AD169 or HCMV Merlin at various MOIs (1–8: MOI 0.5; 13–20: MOI 0.09; 25–32: MOI 0.03; 37–52: MOI 0.01). Cells were fixed within a range of late time points post-infection (1–12: 4 dpi, 13–36: 6 dpi, 37–56: 5 dpi) and immunostained with pAb(r)-cyclin (pAb, polyclonal antibody) T1, mAb UL97/pAb-UL97 (mAb, monoclonal antibody) and mAb-UL44. MOCK, uninfected cells.

In order to study the intracellular localization patterns of pUL97 and cyclin T1, the cell types, HFF, MRC-5, ARPE-19 and TEV-1 were infected with two different HCMV strains, AD169 and Merlin. The results of indirect immunofluorescence analyses are shown in Figure 3. 

Cyclin T1 was found evenly distributed in the nuclei of uninfected cells, regardless of cell type (Figure 3, panels 10, 22, 34, 54). In HCMV-infected cells, however, cyclin T1 was concentrated in subnuclear compartments, partly colocalizing with the viral protein, pUL97 (Figure 3, panels 4, 16, 28, 40, 48). This colocalization was most pronounced in viral replication centres. The kinase pUL97 is a known component of viral replication centres [33], here indicated by co-staining with the viral DNA polymerase processivity factor, pUL44 (Figure 3, panels 6, 18, 30, 42, 50). The pUL97-pUL44 colocalization, previously described in HFFs [33], was confirmed in MRC-5, ARPE-19 and TEV-1 cells. In summary, the subnuclear distribution patterns of pUL97 and cyclin T1 appeared to be rather invariant and did not show detectable differences between cell types and HCMV strains.

### 2.3. Mapping of the Sequence Domain of pUL97 Responsible for the Interaction with Cyclin T1

**Figure 4:**
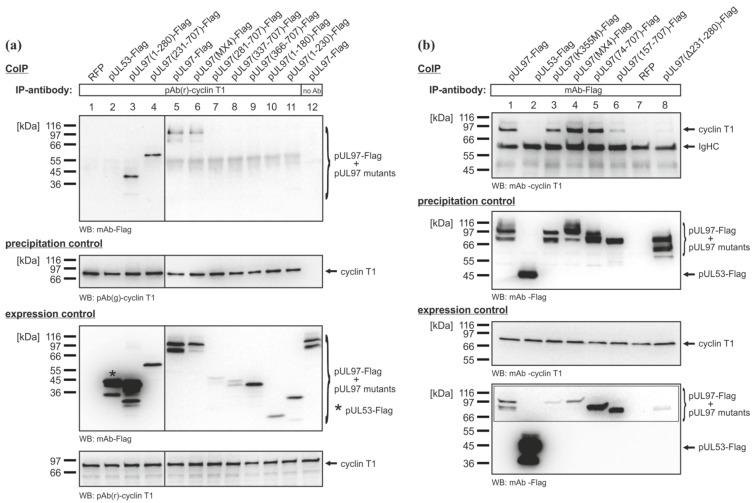
Mapping of the sequence domain of pUL97 responsible for the interaction with cyclin T1. Flag-tagged N- and C-terminal amino acid replacement and deletion mutants of pUL97 were transiently expressed in 293T cells. Recombinantly expressed RFP was used as the transfection control. At two days post-transfection, CoIP analyses were performed. Either (**a**) endogenous cyclin T1 was immunoprecipitated using pAb(r)-cyclin T1 or (**b**) Flag‑tagged pUL97 mutants were immunoprecipitated using mAb-Flag. Specificity of precipitation was demonstrated by CoIP using Dynabeads® Protein A without antibody (**a**, lane 12) and by the negative controls with recombinantly expressed pUL53-Flag (**a** and **b**, lane 2). pUL97(MX4)-Flag, multiple amino acid replacement mutant carrying missense mutations in alternative start codons of pUL97 isoform expression: M38A, M74L, M111L and M157A.

CoIP analyses using Flag-tagged N- and C-terminal truncation mutants of pUL97 expressed in 293T cells were performed to narrow down the sequence domain of pUL97 responsible for the interaction with cyclin T1 (Figure 4a). Initial CoIP analyses revealed that the N-terminus of pUL97 was important for the interaction with cyclin T1 (*i.e.*, amino acids 1–365; data not shown). Further CoIP analyses demonstrated that the first 230 amino acids of pUL97 were dispensable for the interaction with cyclin T1, since pUL97(231–707)-Flag was the smallest N-terminally truncated fragment that was still co-immunoprecipitated, whereas the correlating C-terminal truncation mutant, pUL97(1–230)-Flag, did not interact with cyclin T1 (Figure 4a, lanes 4 and 11). Together with pUL97(1–280)-Flag being the smallest C-terminally truncated pUL97 fragment still interacting with cyclin T (Figure 4a, lane 3), the protein domain of pUL97 responsible for the interaction with cyclin T1 could be mapped to amino acids 231–280. Deletion of the interaction region in construct pUL97(Δ231–280)-Flag prevented the co-immunoprecipitation of cyclin T1, confirming the finding of the mapping experiment (Figure 4b, lane 8). All three isoforms of pUL97 contain the cyclin T1 interaction region and interacted with cyclin T1, as shown by CoIP analyses performed with expression constructs encoding individual isoforms (Figure 4b, lane 4 (IF-1, pUL97(MX4)-Flag), lane 5 (IF-2 and IF-3, pUL97(74–707)-Flag) and lane 6 (IF-3, pUL97(157–707)-Flag) [25,26]). It should be mentioned that in CoIP analyses based on HCMV-infected cell lysates, co-immunoprecipitation of isoform 1 with cyclin T1 was mostly detected (Figure 2c, lanes 2 and 3), which, however, might rather refer to a limitation in detection sensitivity than to biochemical differences between pUL97 isoforms expressed in the two systems. Interestingly, the catalytically inactive mutant, pUL97(K355M)-Flag, also interacted with cyclin T1 (Figure 4b, lane 3), suggesting a mode of pUL97-cyclin T1 interaction independent of pUL97 activity.

### 2.4. Interaction of the HCMV Protein Kinase pUL97 with Further Cyclins

**Figure 5:**
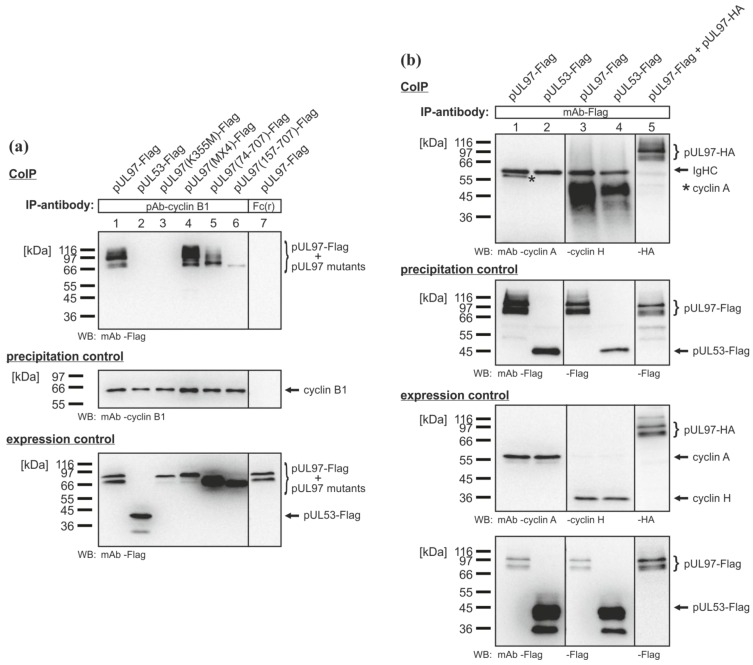
CoIP analyses used to reveal further cyclin interaction partners of pUL97. Flag‑tagged amino acid replacement and deletion mutants of pUL97 were transiently expressed in 293T cells. At 2 days post-transfection, cells were lysed and used for co-immunoprecipitation analysis with (**a**) pAb-cyclin B1 (lanes 1–6) and the Fc fragments of rabbit antibodies as the control (Fc(r), Rabbit IgG Fc-Fragment, Jackson ImmunoResearch; lane 7), respectively; or with (**b**) mAb-Flag. CoIP analyses with recombinantly expressed pUL53-Flag (**a**, lane 2; **b**, lanes 2 and 4) served as a negative control and the self-interaction of pUL97 as the positive control (**b**, lane 5). * cyclin A-specific band.

Further analysis revealed that pUL97 also interacted with cyclins other than cyclin T1. pUL97 was detected in precipitates of cyclin B1, the regulatory cofactor of CDK1, in a CoIP analysis using protein lysates of transiently transfected 293T cells (Figure 5a, lane 1). Like cyclin T1, cyclin B1 also interacted with the three isoforms of pUL97 (Figure 5a, lanes 4 (IF-1), 5 (IF-2 and IF-3), 6 (IF-3)). Importantly, the catalytically inactive mutant, pUL97(K355M)-Flag, was not co-immunoprecipitated with cyclin B1 (Figure 5a, lane 3). This finding may suggest that the interaction of pUL97 with cyclin B1 is dependent on the kinase activity of pUL97, in contrast to the interaction with cyclin T1. This points to a mechanistic difference between pUL97-cyclin T1 and pUL97-cyclin B1 interactions. Additional CoIP experiments also indicated an interaction between pUL97 and cyclin A (regulatory cofactor of CDK1 and CDK2; Figure 5b, lane 1), albeit with a lower affinity, whereas cyclin H (a component of the CDK7/cyclin H/MAT1 complex) could not be identified as an interaction partner of pUL97 (Figure 5b, lane 3). These findings strongly suggest that pUL97 does not exclusively interact with cyclin T1, but additionally with a selection of further cyclins.

### 2.5. Phosphorylation of pUL97 by the CDK9/cyclin T1 Complex in an ATP Consumption Assay

**Figure 6:**
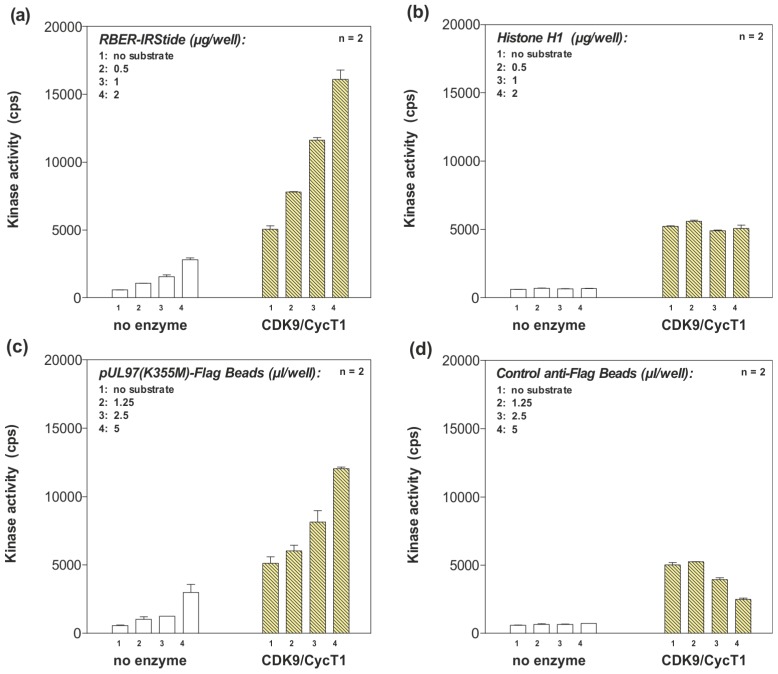
ATP consumption assay used to investigate the putative phosphorylation of pUL97 by the CDK9/cyclin T1 complex. The catalytically inactive mutant pUL97(K355M)-Flag was expressed in 293T cells and 2 days post-transfection, immunoprecipitated from cell lysates using mAb-Flag. Immunoprecipitates and mAb‑Flag coated protein A sepharose beads (negative control) were used to determine luminescence signals (counts per second (cps)) as a measure for ADP generation by the active CDK9/cyclin T1 complex; the assay was performed in duplicate (n = 2). (**a**) Specific substrate RBER‑IRStide (positive control); (**b**) substrate bovine histone H1 (negative control); (**c**) pUL97(K355M)-Flag (substrate in question); (**d**) mAb-Flag coated protein A sepharose beads (negative control). All substrates were used in increasing amounts.

With pUL97 interacting with cyclin T1, we next examined whether pUL97-cyclin T1 interaction triggered phosphorylation of pUL97 by the CDK9/cyclin T1 complex. Sequence analysis of pUL97 revealed that the mapped cyclin T1 interaction region (amino acids 231–280) did not contain the known, linear cyclin recognition motif, DOC_CYCLIN_1 ([RK].L.{0,1}[FYLIVMP]; ELM database [38]), which is found in many CDK substrates. Furthermore, no known CDK phosphorylation site (MOD_CDK_1; ...([ST])P.[KR]; ELM database)) was detected in the amino acid sequence of pUL97. In order to address the question whether pUL97 is phosphorylated by the CDK9/cyclin T1 complex, an ATP consumption assay (ADP-Glo™ Kinase Assay, Promega, Mannheim, Germany) was performed (ProQinase, Freiburg, Germany). In this nonradioactive *in vitro* assay, ATP consumption of CDK9 was measured as an indirect indicator of CDK9 substrate phosphorylation. Importantly, the results strongly indicate phosphorylation of the catalytically inactive mutant, pUL97(K355M)-Flag, by CDK9/cyclin T1 in a substrate concentration-dependent manner (Figure 6c). The detected signals were clearly increased compared to a background control (mAb-Flag (mAb, monoclonal antibody) coated protein A sepharose beads; Figure 6d). Interestingly, an inhibitory, concentration-dependent effect of the control mAb-Flag coated protein A sepharose beads on CDK9 was observed (Figure 6d). If it were the case that CDK9/cyclin T1 was generally inhibited in the presence of protein A sepharose beads, the actual phosphorylation of pUL97(K355M)-Flag might be underestimated under these experimental conditions. RBER-IRStide (artificial fusion protein containing a fragment of human retinoblastoma protein 1, Figure 6a) served as the reference substrate to control the kinase activity of the CDK9/cyclin T1 complex. Bovine histone H1 (a substrate of multiple kinases, excluding CDK9) was used as a negative control to monitor background signals (Figure 6b). CDK9 autophosphorylation signals determined in the absence of substrates were clearly lower than substrate phosphorylation signals (Figure 6a–d, column 1), and substrate background signals measured in the absence of the CDK9/cyclin T1 complex remained at a very low level, close to the detection limit (Figure 6a–d, panel no enzyme). These findings strongly suggest that pUL97 is phosphorylated by the CDK9/cyclin T1 complex. Considering the capability of pUL97 to interact with cyclins other than cyclin T1, the phosphorylation of pUL97 by further CDK/cyclin complexes seems also possible.

## 3. Experimental Section

### 3.1. Cell Culture, HCMV Infections and Plasmid Transfection

Embryonic kidney epithelial cells (293T) and retinal pigment epithelial cells (ARPE-19) were cultivated in Dulbecco’s modified Eagle’s medium containing 10% FCS. Primary human foreskin fibroblasts (HFFs) and human foetal lung fibroblasts (MRC-5) were cultivated in MEM containing 7.5% or 10% FCS, respectively. Human first-trimester extravillous trophoblast (TEV-1) were cultivated in Ham’s F 10 Nutrient Mix supplemented with 10% FCS. The HCMV infection experiments were performed at a multiplicity of infection (m.o.i.) of 1.0 (or lower as indicated for specific experiments) using HCMV strains AD169, AD169-GFP [39] and Merlin [40]. Transfection of 293T cells was performed using polyethylenimine reagent (Sigma-Aldrich, Taufkirchen, Germany), as described previously [29].

### 3.2. Antibodies

The following polyclonal (pAb) and monoclonal (mAb) antibodies were used in the present study: mAb-UL44 (BS 510, kindly provided by Prof. B. Plachter, Univ. Mainz, Mainz, Germany), mAb-UL97 (AL‑1, kindly provided by Prof. M. N. Prichard, Univ. Alabama, Birmingham, AL, USA), pAb-UL97 (06/09, kindly provided by Prof. D. M. Coen, Harvard Medical School, Boston, MA, USA), mAb-pUL69 [41], mAb-cyclin A (sc-271645), mAb-cyclin B1 (sc-7393), pAb-cyclin B1 (sc-752), mAb-cyclin H (sc-1662), mAb-cyclin T1 (sc-271348), pAb(r)-cyclin T1 (sc-10750), pAb(g)-cyclin T1 (sc-8127), mAb-CDK9 (sc-13130; all Santa Cruz Biotechnology, Heidelberg, Germany), mAb-Flag (Sigma-Aldrich, Heidelberg, Germany) and mAb-HA (12CA5, Roche, Mannheim, Germany). The following fluorescent dye-conjugated secondary antibodies were applied in immunofluorescence analyses: Alexa 488-conjugated goat anti-rabbit IgG (H + L) and Alexa 555‑conjugated goat anti-mouse IgG (H + L) (Life Technologies, Darmstadt, Germany).

### 3.3. Co-Immunoprecipitation

293T cells were seeded in 10-cm dishes (cell number: 5.1 × 10^6^) and transfected with expression plasmids coding for full-length pUL97 or mutants of pUL97 and pUL53, as described previously [23,26,29,42] using the polyethylenimine transfection technique [29]. Red fluorescent protein (RFP; pDsRed1-N1; BD Clontech, Heidelberg, Germany) was used as a transfection control. HCMV AD169-GFP-infected HFFs cultivated in cell culture flasks were harvested at 6 days post-infection. Both transfected 293T cells and HCMV infected HFFs were lysed in 500 mL CoIP buffer (50 mM Tris/HCl (pH 8.0), 150 mM NaCl, 5 mM EDTA, 0.5% NP-40, 1 mM PMSF, 2 µg aprotinin mL^−1^, 2 µg leupeptin mL^−1^ and 2 µg pepstatin mL^−1^) and incubated with antibody-coated Dynabeads® Protein A (50 µL per sample; Life Technologies, Darmstadt, Germany) for 2 h at 4 °C under rotation. The precipitates were washed five times (1 mL each) with CoIP buffer before the samples were subjected to standard Western blot analysis using tag- or protein-specific antibodies for the detection of co-immunoprecipitates and protein expression (ECL staining; New England Biolabs, Frankfurt/Main, Germany).

### 3.4. Immunofluorescence Analyses

HFF, MRC-5, ARPE-19 and TEV-1 cells were grown on coverslips and used for infection with various HCMV strains. At indicated time points, cells were fixed with 4% paraformaldehyde solution (10 min, room temperature) and permeabilized by incubation with 0.2% Triton X-100 solution (20 min, 4 °C). Non-specific staining was blocked by incubation with 2 mg/mL human γ-globulin (Cohn fraction II; Sigma Aldrich, Taufkirchen, Germany, 30 min, 37 °C). Proteins were detected by incubation with primary antibodies for 90 min at 37 °C, followed by incubation with dye-conjugated secondary antibodies for 30 min at 37 °C. Cell samples were mounted with Vectashield Mounting Medium containing DAPI and analysed using a DMI6000 B microscope and a 63× HCX PL APO CS oil immersion objective lens (Leica Microsystems, Mannheim, Germany). Confocal laser-scanning microscopy was performed with a TCS SP5 microscope (Leica Microsystems, Mannheim, Germany). Images were processed using the Meta-Imaging series (Molecular Devices, Biberach, Germany), LAS AF software (version 1.8.2 build 1,465; Leica Microsystems) and Adobe Photoshop (version 8.0.1; Adobe Systems Incorporated).

### 3.5. ATP Consumption Assay

An ATP consumption assay (ADP-Glo™ Kinase Assay, Promega, Mannheim, Germany) was performed in order to measure the CDK9-dependent phosphorylation of pUL97 (ProQinase, Freiburg, Germany). The catalytically inactive mutant, pUL97(K355M)-Flag, was expressed in 293T cells. Two days post-transfection, 293T cells were harvested and lysed in 400 µL of RIPA lysis buffer (0.1% SDS, 1% Na‑deoxycholate, 1% Triton X-100, 0.5% NP40, 1 mM EDTA, 10 mM Tris/HCl (pH 7.5), 150 mM NaCl) supplemented with protease inhibitors (Complete Mini, Roche; 1 tablet for 10 mL). The lysate was incubated with mAb-Flag-coated protein A sepharose beads (200 µL of antibody-coated protein A sepharose beads per sample) for 2 h under rotation. The immunoprecipitates were washed with RIPA lysis buffer three times. Washed mAb-Flag coated protein A sepharose beads, incubated with protein lysates from 293T cells not expressing pUL97(K355M)-Flag, were prepared as a negative control. The ATP consumption assay was performed as follows. The reaction cocktail, containing 7.5 μL of 3.33× standard ATP consumption assay buffer (167 mM HEPES-NaOH [pH 7.5], 10 mM MgCl_2_, 10 µM Na‑orthovanadate and 3.33 mM DTT), 7.5 μL of a substrate (various amounts of RBER-IRStide (ProQinase, Freiburg, Germany), histone H1 (Sigma-Aldrich, Munich, Germany), pUL97(K355M)‑Flag immunoprecipitates or mAb-Flag coated protein A sepharose beads), 5 µL of the purified kinase complex, CDK9/cyclin T1 (100 ng diluted in 1× kinase dilution buffer (50 mM HEPES‑NaOH (pH 7.5), 0.25 mg/mL PEG20000, 1 mM DTT), ProQinase, Freiburg, Germany) and 5 μL of ATP (UltraPure), was mixed in 96-well white flat bottom half-area plates and incubated for 30 min at 30 °C (measured in duplicate). Subsequently, 25 µL of ADP-Glo^TM^ reagent were added, and the samples were incubated for 40 min at room temperature. After the addition of 50 µL of ADP‑Glo^TM^ Kinase Detection reagent, the samples were incubated for another 60 min at room temperature in the dark. Resulting luminescence (counts per second (cps)) as a measure for ADP‑generation by the active kinases was determined using a VICTOR reader (Perkin-Elmer, Hamburg, Germany).

### 3.6. Yeast-Two-Hybrid System

Protein interactions were analysed using GAL4 fusion proteins (GAL4-BD, DNA binding domain; GAL4-AD, activation domain) in the yeast two-hybrid system, as described previously [9,34]. pUL97 fused to the GAL4-BD [33,34] and cyclin T1 fused to the GAL4-AD (kindly provided by Dr. Giuliana Napolitano, University of Monte S. Angelo, Italy [43]) were expressed in the Saccharomyces cerevisiae strain, Y153. pGAD424 and pGBT9 (Clontech) served as vector controls and pVA3 (p53) and pTD1 (SV40 large T antigen) as an interaction control. Selection for the presence of bait and interactor plasmids was achieved by cultivation on medium restricting growth to histidine/tryptophan/leucine prototrophy. Selected colonies were analysed for β-galactosidase activity by filter lift tests.

## 4. Conclusions

Interactions between herpesviral kinases and cellular cyclins have not been described so far. This study provides the first evidence that the HCMV protein kinase, pUL97, interacts with cyclins, as demonstrated by well-established methods of yeast two-hybrid assay, co-immunoprecipitation and confocal microscopy-based colocalization studies. In particular, the sequence domain of pUL97, responsible for the interaction with cyclin T1, was between amino acids 231–280. This biochemically defined interaction region is located within the non-globular N-terminus of pUL97 and is distinct from the pUL97 protein kinase domain, which is determined by subdomains I–XI comprising amino acids 337–651 [18]. 

Although this cyclin T1 interaction region of pUL97 does not contain a known cyclin recognition motif and the entire pUL97 sequence does not comprise a known CDK phosphorylation motif, the results of an ATP consumption assay strongly suggest that pUL97 is recognized and phosphorylated by the CDK9/cyclin T1 complex. Due to the fact that kinase activity of CDK9/cyclin T1 was determined in an indirect approach by the quantitative determination of ATP consumption, additional *in vitro* kinase assays using radioactive ATP should be performed to demonstrate the phosphorylation of pUL97 directly and to confirm our conclusion. The detection of further cyclins (cyclin B1 and cyclin A) as interaction partners of pUL97 points towards a more complex mode of pUL97-CDK/cyclin inter-regulation. The question about whether CDK-mediated phosphorylation of pUL97 influences its kinase activity or multifunctionality has not been addressed and requires further investigation. Presently, we are working on the identification of motifs in pUL97 mediating cyclin interaction, such as a putative zinc finger motif (see the interaction between cyclin T1 and that Tat protein of the human immunodeficiency virus [44]) or potential linear interaction motifs [45].

Importantly, the structural and functional similarities between pUL97 and CDKs, the phosphorylation of identical viral and cellular substrates [3] and the interaction between pUL97 and cyclins described here suggest that multimeric complexes may be formed. Such complexes might consist of CDKs, cyclins, pUL97 and further viral proteins or substrates. pUL97 could only transiently be included, so that varying kinase activities might be involved in the regulation of phosphorylation-dependent processes during HCMV replication. They also may only form under certain conditions, as we showed that pUL97 did not co-immunoprecipitate with CDK9. This finding might indicate the lack of interaction or, more likely, a competitive mode of binding of CDK9 and pUL97 to cyclin T1 or the existence of a dynamic complex (trimeric or multimeric) that may only transiently include CDK9, pUL97 and cyclin T1. In the latter scenario, cyclin T1 may play the role of a bridging factor between pUL97 and CDK9, triggering phosphorylation events (see Section 2.5). Interactive regulatory steps of mutual phosphorylation between CDK/cyclin complexes and pUL97 seem probable in this scenario. The regulation of the activity of the viral RNA transporter, pUL69, is an example of such interplay between CDKs and pUL97. In this case, CDK9 and pUL97 contribute to an activating phosphorylation of pUL69 [9,32,46]. The protein region of pUL97 required for the interaction with pUL69 (amino acids 231–336 [32]) overlaps with the cyclin T1 interaction domain of pUL97 (amino acids 231–280), most likely indicating a putative role of cyclin T1 in pUL97-pUL69 interaction. This example of activating pUL69 phosphorylation emphasizes an interplay between CDK and pUL97 activities in phosphorylation-dependent processes during HCMV replication. Interestingly, the mapped region of pUL97 interacting with cyclin T1 is also responsible for the self-interaction of pUL97 (amino acids 231–280 [29]), so that the potential of pUL97 to oligomerize might also be linked to the formation of multimeric complexes. Future experimentation should substantiate this scenario, possibly also including further aspects of pUL97-cyclin interaction, such as a putative regulatory impact on viral DNA synthesis and late gene expression. Taken together, our present and previous findings support the hypothesis that pUL97 not only mimics CDK function, but actively associates with cyclins or CDK/cyclin heteromers to form functional complexes. Thus, the formation of such cyclin-based complexes may provide a platform for pUL97 and CDKs to undergo mutual trans-phosphorylation and fine-regulation.
